# Alcohol-induced apoptosis of oligodendrocytes in the fetal macaque brain

**DOI:** 10.1186/2051-5960-1-23

**Published:** 2013-06-12

**Authors:** Catherine E Creeley, Krikor T Dikranian, Stephen A Johnson, Nuri B Farber, John W Olney

**Affiliations:** 1 Departments of Psychiatry, Washington University School of Medicine, St. Louis, MO 63110, USA; 2 Departments of Pathology, Washington University School of Medicine, St. Louis, MO, USA; 3 Departments of Anatomy and Neurobiology, Washington University School of Medicine, St. Louis, MO, USA

**Keywords:** Alcohol, Apoptosis, Oligodendrocyte, Fetal monkey, Brain

## Abstract

**Background:**

*In utero* exposure of the fetal non-human primate (NHP) brain to alcohol on a single occasion during early or late third-trimester gestation triggers widespread acute apoptotic death of cells in both gray and white matter (WM) regions of the fetal brain. In a prior publication, we documented that the dying gray matter cells are neurons, and described the regional distribution and magnitude of this cell death response. Here, we present new findings regarding the magnitude, identity and maturational status of the dying WM cells in these alcohol-exposed fetal NHP brains.

**Results:**

Our findings document that the dying WM cells belong to the oligodendrocyte (OL) lineage. OLs become vulnerable when they are just beginning to generate myelin basic protein in preparation for myelinating axons, and they remain vulnerable throughout later stages of myelination. We found no evidence linking astrocytes, microglia or OL progenitors to this WM cell death response. The mean density (profiles per mm^3^) of dying WM cells in alcohol-exposed brains was 12.7 times higher than the mean density of WM cells dying by natural apoptosis in drug-naive control brains.

**Conclusions:**

*In utero* exposure of the fetal NHP brain to alcohol on a single occasion triggers widespread acute apoptotic death of neurons (previous study) and of OLs (present study) throughout WM regions of the developing brain. The rate of OL apoptosis in alcohol-exposed brains was 12.7 times higher than the natural OL apoptosis rate. OLs become sensitive to the apoptogenic action of alcohol when they are just beginning to generate constituents of myelin in their cytoplasm, and they remain vulnerable throughout later stages of myelination. There is growing evidence for a similar apoptotic response of both neurons and OLs following exposure of the developing brain to anesthetic and anticonvulsant drugs. Collectively, this body of evidence raises important questions regarding the role that neuro and oligo apoptosis may play in the human condition known as fetal alcohol spectrum disorder (FASD), and also poses a question whether other apoptogenic drugs, although long considered safe for pediatric/obstetric use, may have the potential to cause iatrogenic FASD-like developmental disability syndromes.

## Background

Several classes of drugs, including NMDA antagonists [1], GABA_A_ agonists and alcohol [2,3], which has both NMDA antagonist and GABA agonist properties, trigger widespread neuroapoptosis throughout the developing brain of several animal species [3-11], including non-human primates [12-19]. Included among drugs that have apoptogenic potential are several that are sometimes abused by pregnant women (alcohol, PCP, ketamine, benzodiazepines, barbiturates) and many that are used frequently in pediatric and obstetric medicine (sedatives, anesthetics, anticonvulsants)(reviewed in Creeley and Olney [20]). Evidence documenting neuroapoptosis induced by alcohol and anesthetic drugs is particularly concerning in that millions of human fetuses or infants, every year throughout the world, are exposed to one or more of these agents. The period of peak sensitivity to this neurotoxic mechanism coincides with the brain growth spurt [1,2], during which neurons throughout the brain are undergoing rapid synaptogenesis. In rodents, the brain growth spurt is confined primarily to the first several weeks after birth, but in humans it spans from mid gestation to several years after birth [21]. During this sensitive period, a single drug exposure, at clinically relevant doses, is sufficient to trigger apoptosis of neurons throughout many regions of the brain [3-7,10,11], and cause long-term neurobehavioral disturbances in both rodents [6,20-22] and NHPs [16].

The human relevance of the above findings remains to be fully elucidated. It is well recognized that alcohol can damage the developing human brain and cause a wide variety of neurodevelopmental disabilities referred to as Fetal Alcohol Spectrum Disorders (FASD) [23-25]. Initially, doubts were raised whether anesthetic drugs can cause similar outcomes [26,27], but recently reported evidence from several independent research groups suggests that brief exposure of human infants to anesthesia is associated with increased risk for neurobehavioral disturbances, including learning disabilities and attention deficit/hyperactivity disorder [28-34]. While the original findings pertaining to the apoptogenic action of alcohol and anesthetic drugs on the developing brain were described exclusively in terms of an impact on various neuronal populations, we recently reported that exposure of the neonatal NHP brain to isoflurane [35] or propofol [14] anesthesia triggers a robust apoptotic cell death reaction affecting both neurons and oligodendroglia [OL]. Guerri et al. [36], based primarily on *in vitro* findings, have championed the hypothesis that alcohol might have a toxic effect on astroglia, but OLs have not been suspected of being a target of alcohol’s toxic action. We have reported that exposure of the fetal NHP brain to alcohol on a single occasion can cause widespread death of neurons [15]. In the course of that study, we made the preliminary unreported observation that there was immunohistochemical evidence of a cell death reaction distributed diffusely throughout white matter (WM) regions of these brains. The present study was undertaken to clarify the nature and extent of this toxic reaction to alcohol in WM regions of these alcohol-exposed NHP fetal brains.

## Results

**Figure 1:**
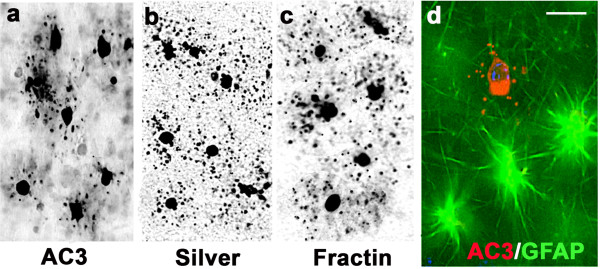
**Histological appearance of apoptotic profiles in frontal subcortical white matter (WM) of alcohol-exposed G120 fetal NHP brain.** Panel (**a**) depicts the appearance of degenerating WM cells stained with antibodies to AC3, and panels (**b**) and (**c**) are from adjacent sections stained with silver or antibodies to fractin. All three of these cell death markers detect a similar number of degenerating profiles in the same WM location, and the dying cells have a similar morphological appearance, regardless whether stained by AC3, silver or fractin. Panel (**d**) displays a WM scene from the same brain immunofluorescently double stained with antibodies to AC3 and GFAP, showing three astrocytes stained with GFAP (green) beside a dying cell stained by AC3 (red). The dying cell is marked by AC3 but not GFAP, signifying that it is not an astrocyte. Scale bar in d = 15 μM for all four panels.

Following up on our preliminary observation that sections from alcohol-exposed fetal NHP brains have abundant AC3-positive profiles distributed widely throughout WM regions [Figure 1a], we stained sections adjacent to the AC3-stained sections with the DeOlmos cupric silver method (marker for dying cells), and observed that approximately the same number of cells in the same WM locations were silver positive [Figure 1b], which provides confirmatory evidence that these WM cells are undergoing cell death. There is evidence that cells in the OL lineage can be stained with antibodies to fractin when they are undergoing apoptotic degeneration [37,38]. Therefore, we stained sections adjacent to those we had stained for AC3 or silver, with antibodies to fractin, and found that fractin staining readily detected a similar number of cells in the same WM locations [Figure 1c], and the appearance of these degenerating WM cells when stained with fractin was very similar to their appearance when stained with AC3 or silver [compare Figure 1a,b,c].

The majority of cells in the WM are either astrocytes or OLs. Therefore, next we performed an immunofluorescent double staining experiment in which AC3-stained sections were also stained with antibodies against GFAP (marker for astrocytes), and found that the AC3-positive profiles did not co-label for GFAP, therefore were not astrocytes [Figure 1d]. In other double staining experiments, consistent with our previously published evidence [35] pertaining to isoflurane-induced apoptosis of OLs, we found that AC3-positive profiles in the WM did not co-label for neuN, PDGFRα, or Iba1, signifying that the vulnerable WM cell is not a neuron, or OL progenitor or a microglial cell.

Collectively, the above findings support the hypothesis that the vulnerable WM cells are OLs that are beyond the progenitor stage of maturation, i.e., OLs that are engaged, or are preparing to engage in myelination. To test this hypothesis we used IHC staining with antibodies to myelin basic protein (MBP) as our primary tool, because MBP IHC provides a means of studying both the myelination process and the role of OLs in mediating that process. We performed MBP staining at several rostrocaudal levels and will present the findings at a rostral level where little or no myelination was occurring, a mid-rostrocaudal level where there was abundant evidence for early myelination, and a caudal level where WM pathways were densely myelinated.

### Findings at a rostral level

**Figure 2:**
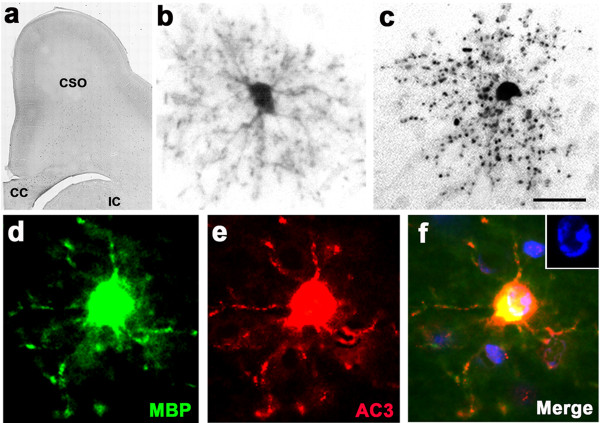
**Section from the rostral forebrain of an alcohol-exposed G120 fetal NHP brain stained with antibodies to MBP, which detects myelinated axons and OLs that contain MBP in their cytoplasm.** Panel (**a**) is a low magnification overview showing that at this rostral level, there is no evidence for myelination of developing axonal tracts coursing through WM regions underlying the frontal cortex (FC), such as the centrum semi-ovale (CSO), corpus callosum (CC), and internal capsule (IC). However, there are many MBP-positive promyelinating OLs visible at higher magnification. Panel (**b**) shows the typical appearance of a normal promyelinating OL that contains enough MBP in its cytoplasm to allow the entire cell to be visualized by MBP staining. At this stage, the OL displays numerous radially oriented processes that have fluffy membranous extensions. Panel (**c**) presents the appearance of an MBP-stained promyelinating OL that is undergoing apoptotic cell death following alcohol exposure. The processes have disintegrated and present as a halo of MBP-positive debris surrounding a condensed cell body. Panels (**d**), (**e**), (**f**) illustrate an MBP-positive promyelinating OL that co-labels with AC3, signifying that it is dying by apoptosis. An abnormal nuclear chromatin pattern revealed by DAPI counterstaining (blue, Fig f inset) confirms the apoptosis diagnosis. Scale bar in (**c**) represents 750 μM in (**a**), 20 μM in (**b**) and (**c**), and 12 μM in (**d-f**).

In sections from the G120 brain cut through the frontal cortex at or rostral to the level of the septum, MBP staining revealed no evidence for myelination [Figure 2a], but at this level there were numerous MBP-positive immature OLs which, judged by their MBP content, were in a state of readiness to begin the myelination process. All of the MBP-positive WM cells at this rostral level had a similar morphology characterized by a dense arbor of radially oriented processes, each of which displayed web-like protoplasmic extensions [Figure 2b]. In alcohol-exposed brains, some of these MBP-positive OLs appeared to be undergoing degeneration, as was evidenced by a halo of particulate debris surrounding the cell body in starburst configuration [Figure 2c], resembling the appearance of degenerating WM profiles stained with silver or fractin [compare Figures 1b and c with 2c]. In double staining experiments it was readily determined that AC3-positive profiles were typically also MBP-positive (Figures 2d,e,f), and DAPI counterstaining revealed an abnormal nuclear chromatin pattern indicative of apoptotic cell death [Figure 2f inset). These findings document that OLs are vulnerable, and onset of vulnerability coincides with the stage when OLs are beginning to synthesize MBP in preparation for myelinating axons. In the literature, OLs at this stage of maturation have been given various designations [37,39,40], depending on the model studied and immunological reagents used. In this writing we will use the nomenclature recommended by Butt and Berry [40] which recognizes a progenitor stage in which the OL cell body stains positive for PDGFRα and negative for MBP. The next stage is the promyelination stage in which the OL cell body becomes positive for MBP and negative for PDGFRα. In this stage the OL is accumulating MBP and other constituents of myelin in its cytoplasm in preparation to begin myelinating axons. When the promyelinating OL begins ensheathing axons and transferring MBP to the sheaths, it is referred to as a myelinating OL, which stains positive for MBP, as do the newly formed myelin sheaths.

### Findings at a mid-rostrocaudal level

**Figure 3:**
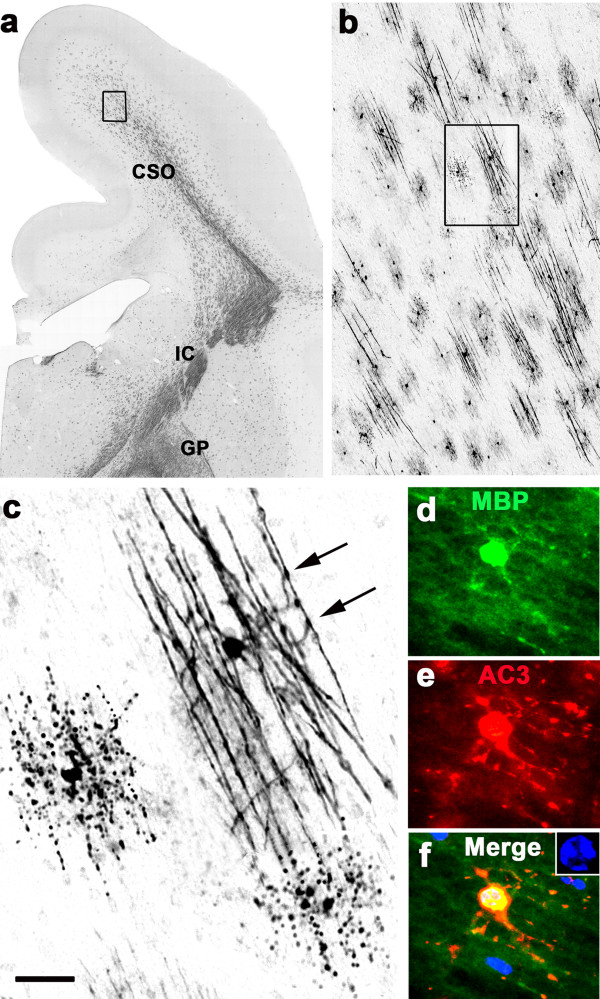
**Section at a mid-rostrocaudal level of an alcohol-exposd G120 fetal NHP brain stained with antibodies to MBP.** Panel (**a**) is an overview showing that several regions at this rostrocaudal level, including centrum semi-ovale (CSO), internal capsule (IC) and globus pallidus (GP), are undergoing myelination. The boxed region in (**a**), shown at higher magnification in (**b**), depicts the typical appearance of early myelination, which is characterized by numerous small patches of axons, each of which is attended by an MBP-positive OL that is actively myelinating that patch. The box in (**b**), magnified in (**c**), shows one such patch consisting of segments of about 10 axons being contacted by the processes of a single OL that is wrapping sheets of MBP-positive membranes around them. Note the intense MBP staining at the points of contact between OL processes and axonal segments (arrows). At the middle left and bottom right of panel (**c**) are MBP-positive degenerating OLs that committed to cell death after having transferred MBP to a patch of axonal segments. Note the linear configuration of the MBP-positive breakdown products that conforms to the layout of an axonal patch. Panels (**d**, **e**, **f**) are double-stained immunofluorescent images documenting the typical appearance of an MBP-positive myelinating OL that co-labels for AC3, signifying that it is undergoing apoptotic cell death, which is confirmed by DAPI counterstaining revealing an abnormal nuclear chromatin pattern (blue, inset panel **f**). Note that the MBP positive processes of this cell contact and appear to be confluent with multple axonal segments, and these same processes are flooded with AC3, even along the surface of the axonal segments. Scale bar in (**c**) represents 1000 μM for (**a**), 100 μM for (**b**), 20 μM for (**c**) and 15 μM for (d,e,f).

In sections from G120 brains at a mid-rostrocaudal level, MBP staining provided evidence for active myelination in several brain regions, including the centrum semi-ovale, corona radiata, internal capsule and globus pallidus [Figure 3a]. At this level, MBP-positive OLs were plentiful, and many were engaged in myelinating axons [Figure 3b & c]. Typically, the early myelination scene featured many small patches of axons undergoing myelination, each patch being attended by a single OL that extended its processes to multiple axonal segments that became visible by virtue of the MBP that the OL was incorporating into their newly formed sheaths [Figure 3b,c]. OLs that were actively myelinating axons, displayed an arbor of multiple slender processes, each of which contacted and appeared to be myelinating several axons. Some of these profiles that were engaged in myelination appeared to be degenerating [Figure 3c]. Interestingly, degenerating OLs in MBP-stained sections presented in two different patterns, depending on the stage of myelination the OL was in when it committed to cell death. If commitment occurred before the OL had effectively transferred its MBP content to axonal processes, the pattern consisted of MBP-positive debris in starburst configuration surrounding a condensed cell body [Figure 2c]. The second pattern consisted of MBP-positive debris distributed in a linear pattern conforming to the layout of an axonal patch [Figure 3c], signifying that the dying OL had committed to cell death after having already myelinated a group of axons. Double staining experiments documented that MBP-positive profiles that were actively engaged in myelination frequently colabeled for AC3 [Figure 3d,e,f], signifying that they were dying by apoptosis, and DAPI counterstaining of these profiles revealed an abnormal nuclear chromatin pattern confirming the apoptosis diagnosis [Figure 3f inset].

### Findings at a caudal level

**Figure 4:**
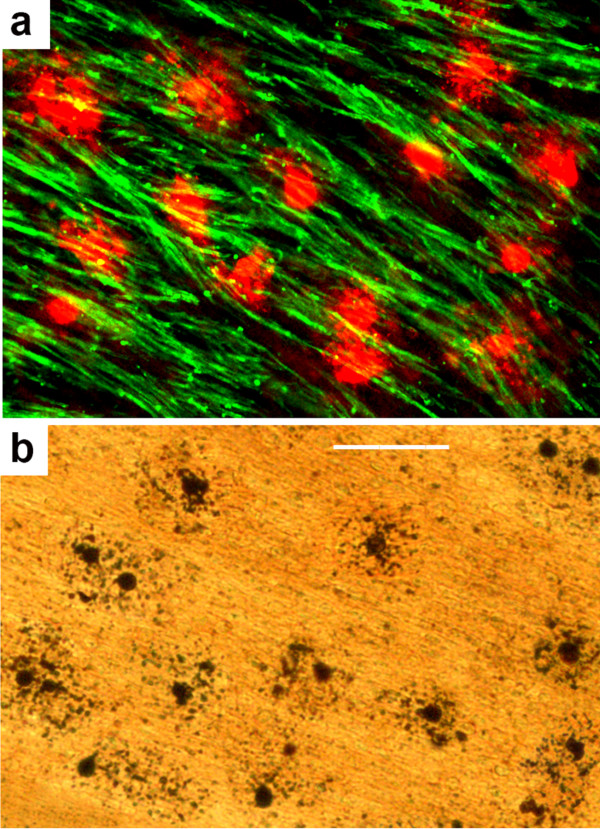
**Scene at a caudal (cerebellar/brainstem) level of an alcohol-exposed G120 fetal NHP brain.** Panels (**a**) and (**b**) are from adjacent sections cut through the cerebellar peduncle. Panel (**a**) is stained immunofluorescently with antibodies to MBP (green) and AC3 (red), and panel (**b**) is stained with the silver method. In (**a**), bundles of heavily myelinated axons are intensely positive for MBP, and numerous cell bodies are intensely positive for AC3, but there are no cell bodies that are stained by MBP. The silver stain in (**b**) detects numerous dying cells that are present in approximately the same density as was revealed by AC3 staining (**a**), which supports the interpretation that these two cell death markers are detecting the same population of dying cells, and they have the same morphological appearance as degenerating OLs in more rostral brain regions. Scale bar in b = 50 μM for both panels.

At a caudal cerebellar/brain stem level, ascending WM tracts were heavily myelinated and MBP staining provided an excellent means of visualizing these myelinated pathways, but MBP did not stain OL cell bodies in these densely myelinated pathways. However, in alcohol-exposed brains, if these densely myelinated pathways were double stained with MBP and AC3, it was possible to detect many cell bodies that were negative for MBP and positive for AC3 amid a dense bed of MBP-positive myelinated axons. In Figure 4a this phenomenon is illustrated in the cerebellar peduncle, a very densely myelinated pathway. Staining of adjacent sections with the silver method (Figure 4b) confirmed that large numbers of cells with OL morphology were undergoing apoptotic cell death in the cerebellar peduncle following alcohol exposure. The observation that two cell death markers identify many dying cells with typical OL morphology in a densely myelinated pathway that has no MBP-positive OL cell bodies, suggests that at this late stage of myelination, the OL cell bodies have ceased generating new MBP and have transferred all of their MBP content to myelin sheaths, which do stain positive for MBP.

The illustrations we have chosen to document the effects of alcohol on the fetal NHP brain are from brain sections of animals at an age early in the third trimester, because this documents that the developing primate brain is vulnerable at the earliest age evaluated in this study, which is an age when human fetuses are frequently exposed to alcohol. We have previously demonstrated [35] that isoflurane anesthesia induces a robust oligoapoptosis response in the primate brain on postnatal day 6. In the present study, the response we observed to alcohol in the G155 fetal brain (1 week before term) was similarly robust (see quantitative evaluation) and showed the same specificity for OL lineage cells at or beyond the maturational stage when they achieve myelination potential. Collectively, our present findings, together with previous findings [35], document vulnerability of the developing primate brain to this toxic mechanism throughout the third trimester of gestation. Thus, exposure of the human fetus to alcohol at any time in the third trimester entails risk of inducing an oligoapoptosis response in the fetal brain.

### Quantitative observations

**Figure 5:**
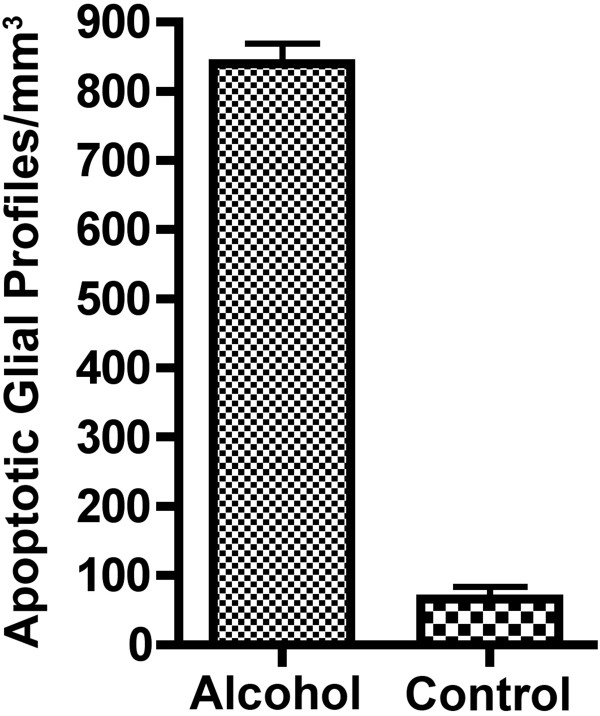
**Mean (± SEM) density of apoptotic OL profiles per mm**^**3 **^**of white matter in the brains of alcohol-exposed (n=5) versus control (n=4) fetal NHPs.** To derive these mean density counts, the following four WM regions were sampled from each brain: the corona radiata, internal capsule, optic tract and cerebellar peduncle.

Quantitative counts performed on WM regions of AC3-stained brain sections revealed that the mean (± SEM) density of apoptotic profiles in the 5 ethanol-exposed brains was 840.3 ± 29.11 profiles/mm3 compared to 66.37 ± 17.97 profiles/mm3 for the 4 control brains (Figure 5). This amounts to a mean 12.7-fold increase in glial apoptotic profiles in the alcohol-exposed compared to the control brains. The difference between the means was 773.9 (95% confidence interval 678.9 to 868.9, p < 0.0001). While the increase in profile density caused by ethanol exposure, when averaged across the three ages, was 12.7 fold, the fold-increase was19.44, 15.27 and 8.45 for the early, mid and late third trimester ages respectively. Thus, the greatest difference between experimental and control density counts was at the earlier ages. Cell death counts based on AC3 staining reveals that the dying OLs in all WM regions at all stages of maturation have a remarkably similar morphology. While the density of degenerating OLs (profiles/mm3) decreased from the early to late gestation period, the toxic impact (in terms of total OLs deleted) remained approximately the same across the different ages because brain mass increases two-fold between the beginning and end of the third trimester. Therefore, if the number of deleted OLs per unit mass of tissue becomes half as many as the tissue mass becomes two times larger, the total number of deleted OLs remains the same.

## Discussion

We have previously reported that a single exposure of the third trimester fetal cynomolgus macaque to alcohol causes widespread apoptotic degeneration of neurons throughout gray matter regions of the developing macaque brain [15]. Here we show in the same group of NHP subjects that alcohol also causes widespread apoptotic degeneration of glial cells throughout WM regions, and the type of glial cell affected is of the oligodendrocyte (OL) lineage. During the NHP third trimester, cells in the OL lineage undergo maturational changes in which OL progenitors differentiate into promyelinating, and then myelinating OLs. During the promyelinating stage they accumulate MBP and other constituents of myelin in their cytoplasm in preparation for myelinating axons. Our evidence indicates that it is early in the promyelination stage that they become vulnerable to alcohol-induced apoptosis, and they continue to be vulnerable throughout both early and late stages of myelination.

An interesting feature of alcohol-induced apoptosis in both the mouse [7] and monkey brain is that the cell death reaction affecting both neurons and OLs is bilaterally symmetrical. Cell death in any region of gray or white matter affecting one hemisphere of an alcohol-exposed brain, is precisely mirrored by the same number and distribution of dying cells among homologous cell populations in the contralateral hemisphere. This feature of alcohol neurotoxicity may significantly limit the capacity for functional recovery, in that the extent of recovery may depend, in part, on the availability of intact contralateral cell populations with similar functional properties to fill in for the cell loss.

It is not clear whether alcohol causes oligoapoptosis by the same mechanism(s) as it causes neuroapoptosis. There is substantial evidence that the neuroapoptotic action of alcohol is triggered by a dual mechanism - blockade of NMDA glutamate receptors and hyperactivation of GABA_A_ receptors [1-3]. Growing evidence for intimate glutamatergic and GABAergic signaling between neurons and OLs via synaptic contacts [41,42] argues in favor of similar cell surface receptor mechanisms triggering these two toxic phenomena. It has been demonstrated that anesthetic drugs with NMDA antagonist or GABA_A_ agonist properties also trigger neuroapoptosis in the developing rodent [1,5,6,43,44] or NHP [12,14,17-19] brain, and that anesthetic drugs also trigger oligoapoptosis in the developing NHP brain [14,35]. Regarding intracellular signaling pathways that may be involved, it has been shown in the *in vivo* mouse brain that this apoptosis process involves Bax-mediated release of cytochrome c from mitochondria which results in activation of caspases 9 and 3 [45-47]. An important upstream event that occurs within minutes after administration of alcohol or anesthetic drugs (prior to Bax protein activation), is the suppression of ERK (extracellular signal-regulated kinase) phosphorylation. Within minutes after administration, both alcohol and anesthetic drugs trigger suppression of pERK, and lithium counteracts this pERK suppressant action, and prevents alcohol or anesthetic drugs from triggering neuroapoptosis [48,49]. It remains to be determined whether these apoptogenic agents suppress ERK phosphorylation in oligodendrocytes, and whether lithium protects against oligoapoptosis induced by these agents.

The neurodevelopmental disabilities associated with FASD are heterogeneous; in childhood, they vary from mild attention deficit/hyperactivity disorder (AD/HD) and related learning disturbances to frank mental retardation [24,25]. Later in life, individuals with a FASD diagnosis have a high incidence of adult-onset neuropsychiatric disturbances, including a 40% incidence of psychosis and 44% incidence of major depressive disorder [23]. The observation that alcohol deletes glia, as well as neurons, from the developing primate brain, and that the glial cell type affected is of the OL lineage, strengthens the proposal that the apoptogenic action of alcohol can explain a wide range of FASD neuropsychiatric disturbances. Deletion of OLs at a stage when they are just beginning to myelinate axons that interconnect neurons throughout the developing brain, could add to both the severity and complexity of long term neurobehavioral consequences of alcohol exposure during fetal life.

While the present findings pertain only to alcohol, and have primary significance in relation to FASD, the potential public health significance is extended by mounting evidence that many drugs (sedatives, anesthetics, anti-convulsants) used in obstetric and pediatric medicine mimic the apoptogenic action of alcohol in the developing brains of several animal species, including NHPs [20]. In addition, a single exposure to these agents can cause long-term neurobehavioral disturbances in several animal species [6,16,43,44,50,51] similar to those caused by alcohol in rodents [22] or in humans [25]. Of potentially greater import is recent evidence reported by several independent research groups [28-34] suggesting that brief exposure of human infants to anesthesia is associated with increased risk for FASD-like neurobehavioral disturbances, including learning disabilities and AD/HD. It was found in several of these studies that increased risk for neurobehavioral disturbances was positively correlated with an increased number (or total duration) of exposures to anesthesia. These observations suggest an explanation why alcohol causes more conspicuous or readily detected neuropsychiatric disturbances than other apoptogenic agents; i.e., it is more common for the developing human brain to be exposed on multiple occasions during critical stages of development to alcohol than to other apoptogenic drugs. Also relevant to the multiple exposure hypothesis, we have found [3] that valproate is one of the most potent anticonvulsants in triggering neuroapoptosis in the infant rat brain, and there is evidence from recent multicenter studies [52,53] that daily exposure of human fetuses to valproate during the third trimester of pregnancy is associated with a 9 point deficit in IQ, and that multiple third trimester exposures to other anticonvulsant drugs ((carbamazepine, lamotrigine, phenytoin) is associated with impaired verbal abilities at age 4.5 years.

Our findings document that OLs become vulnerable to alcohol-induced apoptosis at a maturational stage when they are just beginning to generate MBP in preparation to myelinate axons. At each developmental age evaluated (early, mid, late third trimester) there were abundant OLs at or beyond this maturational stage. This signifies that the developing NHP brain is vulnerable to alcohol-induced oligoapoptosis throughout the entire third trimester of gestation. Early research focusing on alcohol-induced apoptosis of neurons established that the window of vulnerability for that phenomenon coincided with the brain growth spurt period when billions of recently differentiated neurons are expanding their dendritic surfaces to accommodate incoming synaptic contacts. Thus, vulnerability of neurons appeared to be linked to the period of rapid synaptogenesis. Our present data suggest that vulnerability of OLs is linked to a different functional parameter - myelinogenesis. Therefore, it is possible, indeed likely, that the window of vulnerability for alcohol-induced oligoapoptosis will be found to have a different time schedule - one that corresponds to the progression of myelination events and to the maturational status of OLs that are responsible for the generation and maintenance of myelin. Our present findings indicate that onset of vulnerability to the apoptogenic action of alcohol corresponds not to the time when myelin sheaths are formed, but rather to the earlier time when promyelinating OLs are beginning to generate constituents of myelin, which in the human developing brain may occur 2–3 months earlier than myelination of axons is scheduled to begin [54].

The finding that alcohol and anesthetic drugs cause degeneration of OLs in the WM of the developing fetal primate brain raises a question how this toxic reaction might relate to another form of WM injury, periventricular leukomalacia (PVL), which occurs frequently in the brains of prematurely born human infants and results in cerebral palsy. The mechanism(s) underlying PVL are poorly understood, but hypoxia is a leading candidate, and the pathological reaction is thought to involve death of both neurons and OLs [55]. However, in animal research pertaining to hypoxic brain injury, the evidence suggests that death of both neurons and OLs is triggered by an excitotoxic (not apoptotic) mechanism, and OLs that are preferentially killed have been identified as OL progenitors that have not yet begun to generate constituents of myelin in their cytoplasm [37,56,57]. Another distinction is that OL degeneration in PVL is focally concentrated in periventricular regions, whereas OL apoptosis induced by alcohol or anesthetic drugs is diffusely distributed throughout WM pathways. Therefore, these are apparently two distinctly different forms of WM injury. However, if the third trimester brain were exposed to alcohol or anesthetic drugs and hypoxic conditions at the same time, the two forms of pathology, each involving death of both neurons and OLs, would undoubtedly act in concert to increase the severity of brain injury and long-term neurodevelopmental disability. Unfortunately, in pediatric medicine there are settings that are particularly conducive to this type of compound neurotoxicity. For example, in neonatal intensive care units throughout the world, premature infants are commonly exposed intermittently or continuously for days or weeks to anesthetic drugs for purposes of procedural sedation, and these same infants have a weak respiratory reflex, making them prone to spells of apnea (hypoxia).

An important limitation of our current findings is that they do not shed light on how alcohol exerts toxic effects on the brain during very early stages of development. However, Sulik and colleagues [58,59] have demonstrated that *in utero* exposure of mice to alcohol at early stages of embryogenesis markedly increases the rate of apoptotic cell death among specific embryonic precursor cell populations that are destined to make important contributions to the glial and neuronal makeup of the brain. Collectively, our findings and those of Sulik et al., support a unifying and research-guiding hypothesis that, although apoptotic cell death is a natural phenomenon during development, pathological augmentation of apoptotic cell death is unnatural, and represents an occult mechanism by which alcohol, or other drugs that mimic alcohol’s apoptogenic action, can disrupt CNS development at any point from early embryogenesis to several years after birth.

## Conclusions

*In utero* exposure of the fetal NHP brain to alcohol on a single occasion triggers widespread acute apoptotic death of neurons (previous study) and of OLs (present study) throughout WM regions of the developing brain. The rate of OL apoptosis in alcohol-exposed brains was 12.7 times higher than the natural OL apoptosis rate. OLs become sensitive to the apoptogenic action of alcohol when they are just beginning to generate constituents of myelin in their cytoplasm, and they remain vulnerable throughout later stages of myelination. There is growing evidence for a similar apoptotic response of both neurons and OLs following exposure of the developing brain to anesthetic and anticonvulsant drugs. Collectively, this body of evidence raises important questions regarding the role that neuro and oligo apoptosis may play in the human condition known as fetal alcohol spectrum disorder (FASD), and also poses a question whether other apoptogenic drugs, although long considered safe for pediatric/obstetric use, may have the potential to cause iatrogenic FASD-like developmental disability syndromes.

## Methods

### Animals

The NHP subjects for this research were fascicularis (cynomolgus) macaques supplied by Alpha Genesis Inc., a non-human primate facility in Yamassee, South Carolina. All NHP experimental procedures were conducted at the Alpha Genesis facility under the supervision of one of the authors (NBF) and the Alpha Genesis veterinary staff, and the brains of the fetuses were transported to the authors’ laboratories for histological evaluation. All aspects of the research were approved by the Institutional Animal Care and Use Committees of Alpha Genesis Inc. and Washington University School of Medicine, and were conducted in full accordance with the PHS Policy on Humane Care and Use of Laboratory Animals. In the interests of conserving scarce and precious primate resources, this study was designed to obtain a maximal amount of information from as small a number of NHP fetuses as possible. Thus, the data were obtained from two alcohol-exposed and one control subject at an early third trimester age [gestational age 105 to 120 days (G105-120)], two alcohol-exposed and two control subjects at mid third trimester (G125-140), and one alcohol-exposed and one control at late third trimester (G145-155). The full term gestation period for this macaque specie is 160–165 days [60].

Pregnant female NHPs at the desired gestational age received an intravenous injection of saline (controls) or alcohol (2.15 g/kg) administered over a 2 minute period (time zero). Every hour for 6 hours a maintenance dose (0.2 g/kg) was administered to maintain the blood alcohol concentration (BAC) in the range of 300–400 mg/dl. This blood level was chosen because it approximates the BAC that a human fetus would be exposed to during a moderately heavy maternal binge drinking episode. For example, see Minion et al. [61] who reported that 204 patients seen in an adult emergency room for alcohol intoxication had an average BAC = 467 mg/dl, and some of them had BACs > 600 mg/dl. Others [62] have reported very similar data, and the alcohol literature, in general, documents that individuals who are dependent on alcohol tolerate and crave much higher BACs than alcohol-naive individuals can tolerate [63]. One hour after the last dose of saline or alcohol (*i.e.* 8 hours from time zero), the mother was anesthetized, and the fetus delivered by caesarian section, then immediately euthanized (while still under anesthesia) by perfusion of fixative (4% paraformaldehyde in phosphate buffer). The fetal brains were additionally preserved in the same fixative for one week after which serial sections were cut 70 μm thick in the transverse plane for the forebrain and midbrain and in the sagittal plane for the brainstem and cerebellum.

### Histopathology

The present study was undertaken to follow up on a preliminary observation that in the WM of NHP fetal brains exposed to alcohol there are large numbers of cellular profiles that can be detected by immunohistochemical (IHC) staining with antibodies to activated caspase 3 (AC3), a marker for apoptotic cell death. In order to confirm that these cells were dying we applied the DeOlmos cupric silver stain, which selectively stains cells that are dead or dying [1,64]. We also performed IHC staining with antibodies to fractin, a breakdown product of actin that is generated by the proteolytic action of caspase enzymes in OLs that are undergoing apoptotic degeneration [37,38]. To clarify the identity and maturational status of these AC3-positive WM cells, we used immunofluorescent double staining for AC3 or fractin (markers for apoptosis) and GFAP (marker for astrocytes), Iba1 (marker for microglia and macrophages) [35], platelet-derived growth factor receptor alpha (PDGFRα) (marker for OL progenitors) [35,39,40], myelin basic protein (MBP) (marker for premyelinating and myelinating OLs) [40], and neuN (marker for neurons). In all of the double staining immunofluorescent experiments we counterstained with DAPI, a stain that detects abnormal changes in nuclear chromatin pattern indicative of apoptotic cell death.

Specific staining protocols were as follows: Our DeOlmos cupric silver method and non-fluorescent staining methods for AC3 have been described previously [1,4,6,11,45]. For immunostaining with MBP (1:100; MAB 395; Millipore, Billerica, MA, USA), fractin (1:400; AB3150; Millipore), Iba1 (1:500; 019–18741; Wako Chemicals, Richmond, VA), we used the Vectastain Elite ABC kit with Vector VIP as chromogen (Vector laboratories, Burlingame, CA, USA). Immunofluorescent detection of caspase-mediated cell death employed an activated caspase-3 (AC3) rabbit primary polyclonal antibody (9661B; 1:500; Cell Signaling Technology, Inc., Danvers, MA). Floating sections were incubated overnight at room temperature (RT). After rinsing in PBS (3X5 min) they were incubated for 2 hr at RT with fluorescent goat anti-rabbit Alexa Fluor 555 (1:1000, Invitrogen), rinsed in PBS, and coverslipped with Vectashield mounting medium. Double-staining for AC3 with MBP (1:100, MAB 395, Millipore), NeuN (1:200, MAB 377, Millipore), GFAP (1:500; Mab360; Millipore) was accomplished using complementary fluorescent secondary antibodies (1:1000, Alexa Fluor 488). Double-staining for AC3 with PDGFRα employed a goat anti PDGFRα antibody (1:6; AF-307-NA; R & D systems; Minneapolis, MN) followed by complementary fluorescent secondary antibodies (1:100 Alexa Fluor 488 and Alexa Fluor 555). Double-staining for Fractin (1:400; AB3150, Millipore) and MBP (1:100; MAB 395, Millipore) was achieved using complementary fluorescent (1:1000; Alexa Fluor 488 and Alexa Fluor 555) secondary antibodies. All immunofluorescent co-labeling experiments included DAPI in the mounting medium (Vectashield, Vector) to clarify whether cellular profiles staining positive for apoptosis markers also showed condensation and clumping of nuclear chromatin, an abnormal pattern indicative of apoptotic cell death.

### Quantitative methods

For quantification of the glial response we sampled four regions of WM, the corona radiata, internal capsule, optic tract and cerebellar peduncle. To sample the four designated regions we used a template of appropriate size and shape to fit over a core portion of each designated area. The same template was used to sample a given region for each age-matched pair, and anatomical landmarks were used to ensure that the same anatomical region was sampled for each member of the pair. Counts were obtained from 3 consecutive sections from each brain region. The sampling procedure was computer assisted with StereoInvestigator software (Microbrightfield Inc., Williston, VT, USA) so that the total volume of tissue sampled from each region in a given pair of alcohol and control brains was controlled at an identical value. The number of AC3 positive profiles from each WM region and the volume of tissue sampled were summed for each brain. Dividing the total volume into the total profile count yielded a density count (profiles per mm^3^) for each alcohol-exposed and each control brain. This provided a quantitative basis for comparing each age-matched alcohol/control pair and also for comparing the total mean density counts for all alcohol-exposed brains combined with the total mean density counts for all control brains combined.

### Statistical analysis

To determine if differences between the experimental and control brains were statistically significant, an unpaired students t test with Welch correction, where appropriate, was performed comparing the total mean (± SEM) density counts for the alcohol brains combined (n = 5) versus the total mean (± SEM) density counts for the control brains combined (n = 4).

## Abbreviations

OL: Oligodendrocyte; WM: White matter; NMDA: N-Methyl-D-aspartate; GABA: Gamma amino butyric acid; NHP: Non-human primate; AC3: Activated caspase 3; MBP: Myelin basic protein; BAC: Blood alcohol concentration; IHC: Immunohistochemistry; GFAP: Glial fibrillary acidic protein; Iba1: Ionized calcium binding adaptor molecule 1; PDGFRα: Platelet-derived growth factor receptor alpha; DAPI: 4′,6-diamidino-2-phenylindole; FASD: Fetal alcohol spectrum disorder.

## Competing interests

The authors do not have any competing or conflicting interests to report.

## Authors’ contributions

JWO, principal investigator of NIH grants that supported this work, was instrumental in overseeing the conduct of the entire study, and participated in research design, data analysis, and manuscript writing; CEC supervised the histopathological processing, staining, and immunofluorescent imaging of brain tissue and contributed to the research design, data analysis and manuscript writing; KTD provided expert assistance in application of immunohistochemical and immunofluorescent methods and in interpretation of the histopathological findings; SAJ assisted in the processing and staining of brain tissue and was responsible for quantitative analysis of apoptotic profiles in the alcohol-exposed and control brains; NBF was responsible for on site supervision of the experimental procedures conducted at the Primate Breeding facility, including administration of alcohol to pregnant NHP dams and delivery of the fetuses by caesarian section. All of the Authors have read and approve of the written manuscript.
